# Serum Irisin as a Potential Biomarker for Cognitive Decline in Vascular Dementia

**DOI:** 10.3389/fneur.2021.755046

**Published:** 2021-09-13

**Authors:** Feng Zhang, Guangshun Hou, Guangjian Hou, Congan Wang, Bin Shi, Yuekun Zheng

**Affiliations:** ^1^Neck-Shoulder and Lumbocrural Pain Hospital of Shandong First Medical University, Shandong First Medical University and Shandong Academy of Medical Sciences, Jinan, China; ^2^Qilu Children's Hospital of Shandong University, Shandong University, Jinan, China

**Keywords:** irisin, biomarker, vascular dementia, cognitive, ELISA

## Abstract

**Background:** Irisin, a new exercise-related myokine, has been shown to be associated with a variety of diseases including serious neurological disorders. However, whether irisin is involved in the pathogenesis of vascular dementia (VD) has not yet been reported. Our aim is to determine the serum irisin level in patients with VD and investigate its relationship with cognitive function.

**Methods:** The subjects of the study were VD patients and controls with normal cognitive function who were hospitalized in the Neck-Shoulder and Lumbocrural Pain Hospital of Shandong First Medical University from July 2018 to June 2020. Upon admission, a cognitive function assessment was performed. Enzyme-linked immunosorbent assay (ELISA) was used to determine the concentration of irisin in serum.

**Results:** During the study period, 187 subjects (82 controls and 105 VD patients) were included in the analysis. The serum irisin level of VD patients was significantly lower than that of the control group (*p* < 0.001). Spearman analysis showed that irisin was positively correlated with HLD-C and MoCA, and negatively correlated with all clinical characteristics except for HCY. Logistic regression analysis showed that after adjusting for all clinical characteristics, the serum irisin of VD patients still had a significant correlation with MoCA (β = 0.304, *p* = 0.029). According to receiver operating characteristic (ROC) curve analysis, the diagnostic accuracy for serum irisin levels on VD was 76% with the sensitivity and 71% with specificity respectively.

**Conclusions:** These data indicate that a decrease in serum irisin levels is a powerful biological marker for cognitive decline in patients with VD, even after adjustment for risk factors. Further multi-center studies need to confirm this connection, which may pave the way for new treatment options.

## Introduction

Vascular dementia (VD) is generally considered to be the second largest subtype of dementia (1, 2). It accounts for about 15–20% of dementia cases in Europe and America, and it is even higher in developing countries, especially in Asia, at about 30% (3). The incidence of VD increases sharply with age, and its prevalence in countries around the world is increasing with the advent of an aging society (4). According to reports, there are currently ~35.6 million people suffering from dementia in the world, and the number of patients will increase by two times and three times by 2030 and 2050, respectively (5, 6). Unfortunately, similar to Alzheimer's disease (AD), the pathogenic mechanism of VD has not yet been fully identified and understood (7). Therefore, identifying the potential pathogenic biomarkers of VD and adopting targeted interventions are public health problems to be solved urgently.

Irisin, first described by Böstrom et al. in 2012, is a small polypeptide with 123 amino acids released into the blood from the membrane protein fibronectin type III domain-containing protein 5 (FNDC5), which is cleaved at the carboxy terminus by protease in skeletal muscle (8). FNDC5 is a well-defined myokine, which is also considered to be an adipokine. FNDC5/irisin axis was activated by exercise and is relatively conserved in evolution across a number of species (9). Although the research on irisin has received extensive attention in the past few years, the cleavage mechanisms of FNDC5 protein and the regulation pathways of irisin are still unclear (10).

Initial studies have shown that the expression of irisin is related to obesity, type 2 diabetes, and metabolic syndrome (11). More studies have shown that irisin is closely related to the prognosis of stroke and affective disorder after stroke (12, 13). However, whether irisin plays a role in the pathogenesis of VD is unclear. Therefore, in this study, we studied the correlation between irisin concentration and cognitive function in VD.

## Methods

### Study Population

**Figure 1:**
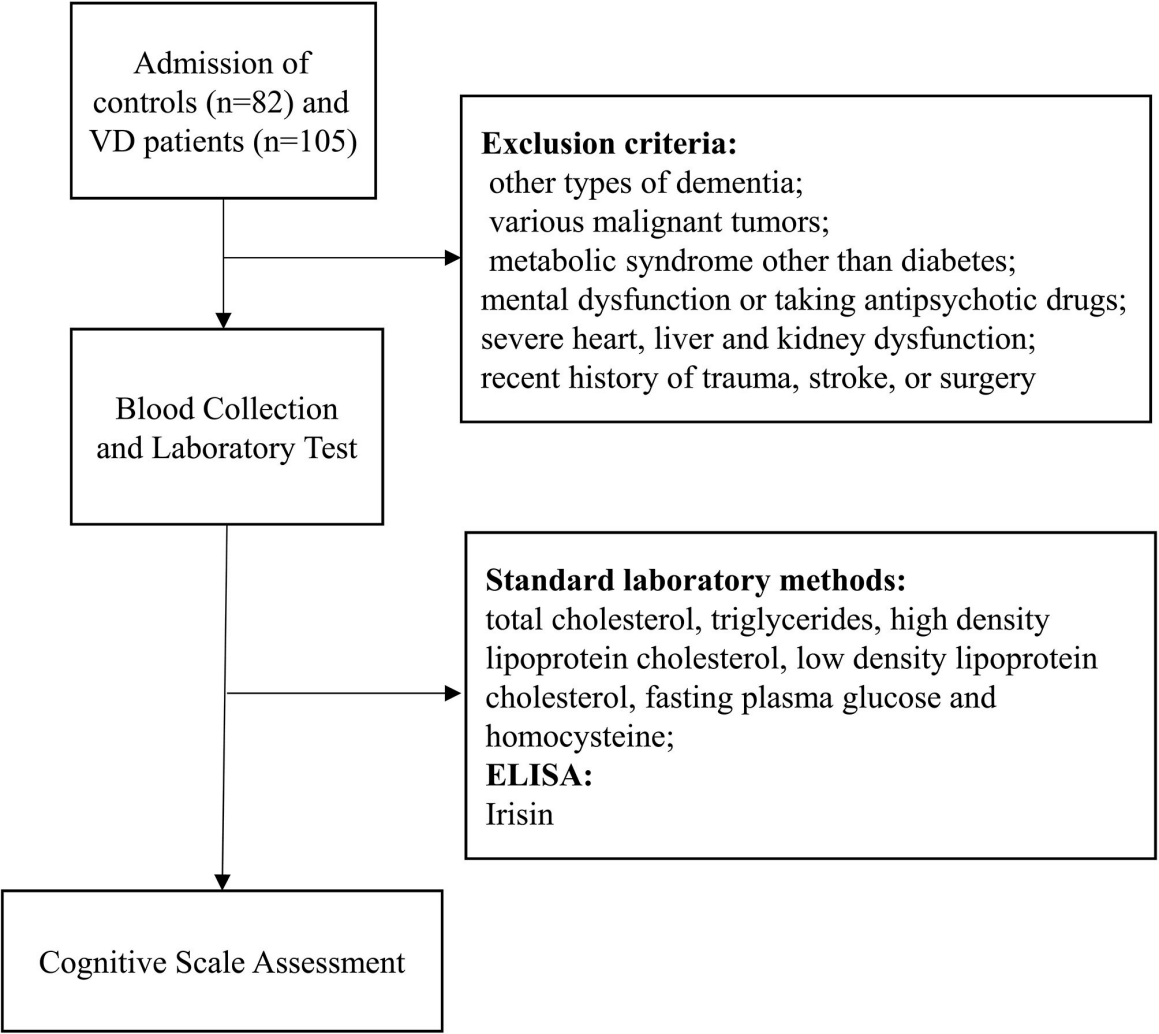
Flow chart of the study implementation.

The study population were VD patients and controls with normal cognitive function who were hospitalized in the Neck-Shoulder and Lumbocrural Pain Hospital of Shandong First Medical University from July 2018 to June 2020 (Figure 1). VD was defined according to the International Statistical Classification of Diseases and Related Health Problems 11 (ICD-11) (14) and the Diagnostic and Statistical Manual of Mental Disorders 5th edition (DSM-5) (15). The exclusion criteria are as follows: (1) previous diagnosis of other types of dementia; (2) various malignant tumors; (3) metabolic syndrome other than diabetes; (4) mental dysfunction or taking antipsychotic drugs; (5) severe heart, liver and kidney dysfunction; (6) recent history of trauma, stroke, or surgery. All research subjects signed an informed consent form. The age, gender, Body Mass Index (BMI) and blood pressure information of all subjects were collected at the time of enrollment. This study complies with the principles of the Declaration of Helsinki and was approved by the Ethics Committee of Neck-Shoulder and Lumbocrural Pain Hospital of Shandong First Medical University.

### Cognitive Scale Assessment

The Montreal Cognitive Assessment (MoCA) is a simple cognitive screening tool, which is widely used in many countries around the world with good psychometric characteristics and excellent sensitivity. MoCA is a 30-point screening tool that takes about 10 min to evaluate attention, orientation, language, memory, visual space, and executive function. The recommended normal range of MoCA is 26–30 points (16, 17). The MoCA scale is evaluated by experienced neurologists, who are blind to the grouping of study subjects and serum biomarkers.

### Blood Collection and Laboratory Test

All blood samples were collected within 24 h of admission in a fasting state and rested for at least 10 min. The blood sample was centrifuged at 1,200 g for 20 min, and the separated serum was aliquoted and stored at −80°C for later use (18, 19). Standard laboratory methods are used for biochemical determination including total cholesterol, triglycerides, high density lipoprotein cholesterol, low density lipoprotein cholesterol, fasting plasma glucose and homocysteine. A commercial enzyme stained immunosorbent assay (ELISA) kit (Aviscera Bioscience, Santa Clara, CA, USA) was used to determine the concentration of irisin in the serum of VD patients. The specific operation was carried out in accordance with the instructions.

### Statistical Analysis

The results of categorical variables are represented by n or percentages, and the results of continuous variables are represented by mean ± standard deviation (SD). Categorical variables are compared by chi-square test, while continuous variables are analyzed by student's *t*-test. Spearman rank correlation was used for bivariate correlation. The relationship between serum irisin concentration and clinical characteristics was evaluated by logistic stepwise regression analysis. Sensitivity and specificity of measured variable for VD diagnosis were determined by receiver operating characteristic (ROC). All statistical analyses were performed using SPSS for Windows, version 23.0 (SPSS Inc., Chicago, IL, USA). Statistical significance is defined as two-tailed *P* < 0.05.

## Results

### Baseline Clinical Characteristics of Subjects

A total of 82 normal cognitive function controls and 105 VD patients were enrolled in this study. The baseline clinical characteristics of all subjects are shown in Table 1. There was no significant difference in baseline data such as age, gender, BMI, SBP, DBP, TC, TG, LDL-C, HDL-C, FBG and HCY between the two groups (*p* > 0.05). The serum irisin concentrations of the control group and the VD group were (127.80 ± 11.15) and (79.37 ± 8.74) ng/ml, respectively, and there are significant differences between the two groups (*p* < 0.001).

**Table 1:** Baseline clinical characteristics of all subjects.

	**Controls (*n* = 82)**	**VD (*n* = 105)**	***P-*values**
Age (years)	61.6 ± 6.2	62.0 ± 7.4	0.695
Male (*n*)	43	60	0.524
BMI (Kg/m^2^)	24.2 ± 1.7	24.1 ± 1.9	0.709
SBP (mmHg)	136.3 ± 8.0	136.9 ± 7.8	0.606
DBP (mmHg)	80.5 ± 4.6	81.4 ± 5.1	0.213
TC (mmol/L)	4.71 ± 0.73	4.73 ± 0.67	0.846
TG (mmol/L)	1.57 ± 0.26	1.59 ± 0.30	0.632
LDL-C (mmol/L)	2.45 ± 0.34	2.51 ± 0.35	0.240
HLD-C (mmol/L)	1.17 ± 0.21	1.14 ± 0.22	0.346
HCY (mmol/L)	12.3 ± 1.2	12.6 ± 1.6	0.159
FBG (mmol/L)	6.27 ± 0.79	6.19 ± 0.83	0.505
Irisin (ng/ml)	127.80 ± 11.15	79.37 ± 8.74	<0.001
MoCA scores (points)	27.9 ± 1.1	22.3 ± 1.6	<0.001

*VD, vascular dementia; BMI, Body Mass Index; SBP, systolic blood pressure; DBP, diastolic blood pressure; TC, total cholesterol; TG, triglycerides; HDL-C, high density lipoprotein cholesterol; LDL-C, low density lipoprotein cholesterol; FBG, fasting plasma glucose; HCY, homocysteine; homocysteine*.

### Spearman's Correlation Test

In the Spearman rank correlation test, the serum irisin of VD patients was significantly negatively correlated with age (*r* = −0.231, *p* < 0.001), gender (*r* = −0.174, *p* = 0.005), BMI (*r* = −0.159, *p* < 0.001), SBP (*r* = −0.254, *p* < 0.001), DBP (*r* = −0.217, *p* < 0.001), TC (*r* = −0.322, *p* = 0.013), TG (*r* = −0.283, *p* < 0.001), LDL-C (*r* = −0.195, *p* < 0.001) and FBG (*r* = −0.170. *p* < 0.001), and positively correlated with HDL-C (*r* = 0.272, *p* < 0.001) and MoCA scores (*r* = 0.316, *p* < 0.001). There was no significant correlation between serum irisin and HCY in patients with VD (*r* = −0.308, *p* = 0.094). The results of the Spearman rank correlation test are shown in Table 2.

**Table 2:** Clinical correlations between irisin and various characteristics in VD.

	**Spearman's correlation coefficient**	***P***
Age (years)	−0.231	<0.001
Gender	−0.174	0.005
BMI (Kg/m^2^)	−0.159	<0.001
SBP (mmHg)	−0.254	<0.001
DBP (mmHg)	−0.217	<0.001
TC (mmol/L)	−0.322	0.013
TG (mmol/L)	−0.283	<0.001
LDL-C (mmol/L)	−0.195	<0.001
HLD-C (mmol/L)	0.272	<0.001
HCY (mmol/L)	−0.308	0.094
FBG (mmol/L)	−0.170	<0.001
MoCA scores (points)	0.316	<0.001

*VD, vascular dementia; BMI, Body Mass Index; SBP, systolic blood pressure; DBP, diastolic blood pressure; TC, total cholesterol; TG, triglycerides; HDL-C, high density lipoprotein cholesterol; LDL-C, low density lipoprotein cholesterol; FBG, fasting plasma glucose; HCY, homocysteine; homocysteine*.

### Logistic Regression Analysis

The logistic regression analysis results are shown in Table 3. After controlling for age, gender and BMI in Model 1, there is a significant correlation between the serum irisin concentration of VD patients and the MoCA score (β = 0.385, *p* < 0.001). After further controlling of SBP, DBP, TC, TG, LDL-C, HDL-C and FBG in Model 2, the serum irisin concentration of VD patients still has a significant correlation with the MoCA score (β = 0.336, *p* = 0.008). HCY is considered to be an important factor affecting cognitive function, we pulled it into Model 3. Finally, after controlling of all baseline clinical characteristics in Model 3, the correlation between serum irisin concentration and MoCA score in VD patients is still significant (β = 0.304, *p* = 0.029).

**Table 3:** Association between irisin and MoCA scores in VD.

	**Regression coefficient**	***P***
Model 1	0.385	<0.001
Model 2	0.336	0.008
Model 3	0.304	0.029

*Model 1: adjusted for age, gender and BMI; Model 2: further adjusted for SBP, DBP, TC, TG, LDL-C, HDL-C and FBG; Model 3: further adjusted for HCY. VD, vascular dementia; BMI, Body Mass Index; SBP, systolic blood pressure; DBP, diastolic blood pressure; TC, total cholesterol; TG, triglycerides; HDL-C, high density lipoprotein cholesterol; LDL-C, low density lipoprotein cholesterol; FBG, fasting plasma glucose; HCY, homocysteine; homocysteine*.

### ROC Curve Analysis

**Figure 2:**
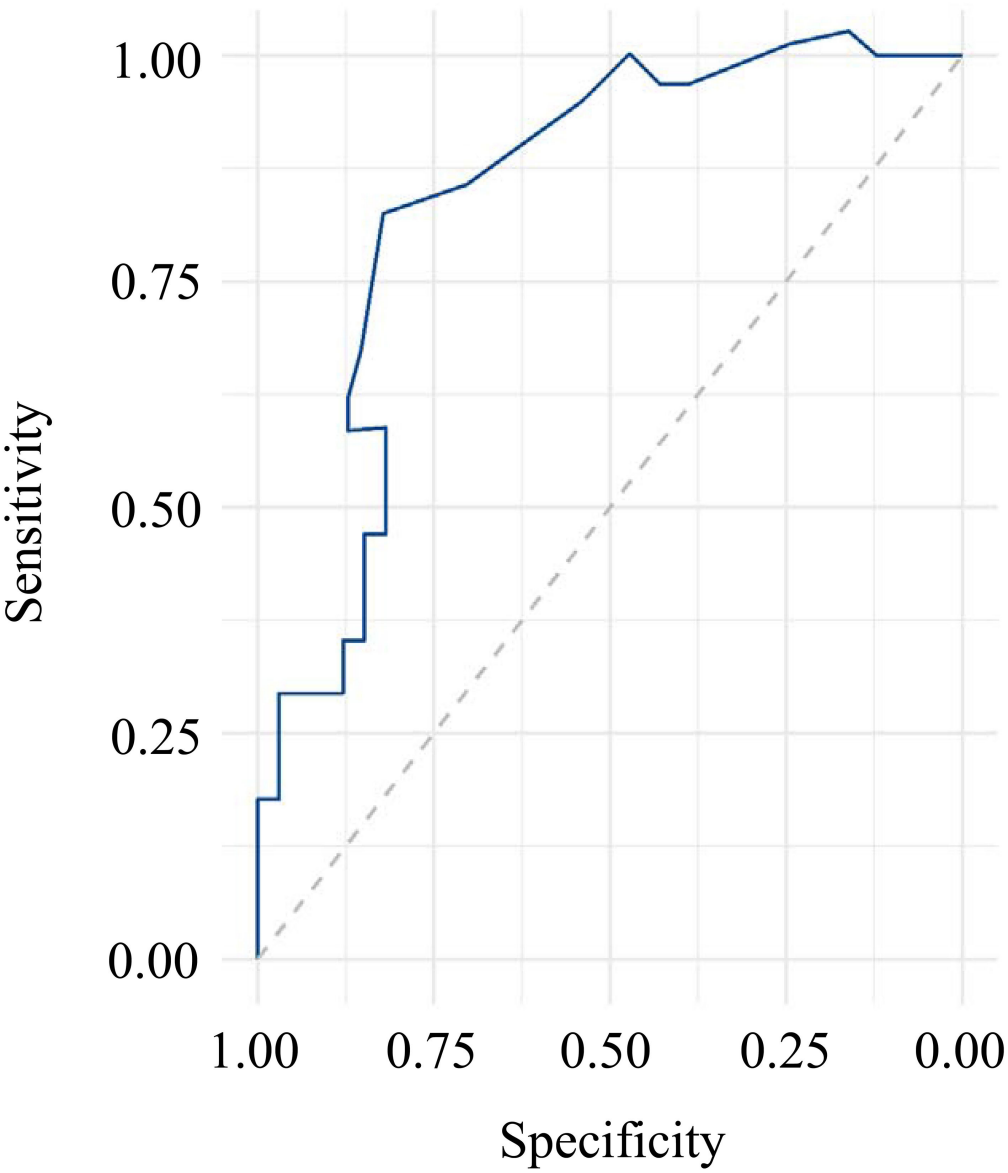
ROC curve analysis for serum irisin levels in all subjects.

To evaluate the diagnostic value of serum irisin levels as a potential marker for VD, ROC curve analysis was performed (Figure 2). The area under the curve (AUC) was 0.846. The diagnostic accuracy for serum irisin levels on VD was 76% with the sensitivity and 71% with specificity, respectively.

## Discussion

This is the first time to study the relationship between cognitive decline and serum irisin concentration in VD patients. Our results show that the serum irisin concentration of VD patients is significantly lower than that of controls with normal cognitive function, and there is a significant positive correlation with the MoCA score. This relationship still exists after adjusting the clinical baseline data, suggesting that serum irisin may be used as a biomarker of cognitive function for VD.

In recent years, irisin has been confirmed to be related to the onset of many diseases. Yeon-Kyung Choi et al. found that serum irisin concentration was lower in newly-onset type 2 diabetes patients and is affected by blood sugar levels, suggesting that irisin played an important role in the pathogenesis of type 2 diabetes (20). The study of Graziana Colaianni et al. showed that irisin could enhance bone mass and improve the strength of skeletal cortex, suggesting that irisin may not only be responsible for the skeletal connection of muscles and bones, but may also become an important means for the treatment of osteoporosis (21). Another study pointed out that serum irisin was a biomarker of metabolic syndrome and cardiovascular disease (22). A study from Taiwan found that the serum irisin level of patients with chronic kidney disease was reduced, and it couldalso affect the cholesterol level and energy metabolism of patients with chronic kidney disease (23).

More and more evidences showed that irisin could also participate in the pathogenesis of a series of neurological diseases. Wen-Jun Tu's team found that serum irisin level was a powerful biological marker for the risk of post-stroke depression (12). The team also found that the decreased serum irisin concentration was a risk factor for the prognosis of acute stroke patients (13). The above two studies have shown that serum irisin is involved in the pathogenesis of acute stroke, but the specific mechanism is still unclear. Irisin is released by the cleavage of the membrane-bound precursor protein FNDC5, which is highly expressed in the hippocampus, while the level of FNDC5/irisin in the AD hippocampus and cerebrospinal fluid is reduced. Therefore, knocking out FNDC5/irisin may impair the cognitive function of mice. On the contrary, overexpression of FNDC5/irisin has neuroprotective effects on synaptic plasticity and memory in mice (24). The protective effect of irisin on AD is also believed to be related to the regulation of inflammation (25). Studies have confirmed that irisin can regulate the release of inflammatory factors in astrocytes and promote the survival of neurons (26). Studies from animal experiments showed that intraperitoneal injection of irisin could increase the expression of neurotrophic genes in the brain of mice and had a certain neuroprotective effect (27). Although irisin is associated with a variety of neurological diseases, there are relatively few reports on its protective effect on cognitive function. The mechanism of irisin involved in cognitive protection deserves further study.

Our research has some limitations. First of all, our research subjects come from a single region and the sample size is relatively small. Secondly, exercise can affect the level of irisin (28). We did not collect information on the subjects' exercise habits in the clinical baseline data. Thirdly, some drugs have potential effects on the level of serum irisin, but we have not collected the patient's medication status. Finally, we did not analyze the relationship between serum irisin and different cognitive domains. Nevertheless, our research is the first study to report the role of irisin in the pathogenesis of VD, which still has great clinical significance.

## Conclusions

This study shows that a decrease in serum irisin level is a potential biological marker of cognitive impairment in VD patients, even after adjustment for confounding factors. Future studies *in vivo* and *in vitro* are needed to further confirm this connection, which may provide a novel treatment for VD.

## Data Availability Statement

The raw data supporting the conclusions of this article will be made available by the authors, without undue reservation.

## Ethics Statement

The studies involving human participants were reviewed and approved by the Ethics Committee of the Neck-Shoulder and Lumbocrural Pain Hospital of Shandong First Medical University. The patients/participants provided written informed consent to participate in this study.

## Author Contributions

YZ designed this research and wrote the manuscript. BS, CW, FZ, GuangjH, and GuangsH participated in data collection, experimental process, and data analysis. All authors contributed to the article and approved the submitted version.

## Conflict of Interest

The authors declare that the research was conducted in the absence of any commercial or financial relationships that could be construed as a potential conflict of interest.

## Publisher's Note

All claims expressed in this article are solely those of the authors and do not necessarily represent those of their affiliated organizations, or those of the publisher, the editors and the reviewers. Any product that may be evaluated in this article, or claim that may be made by its manufacturer, is not guaranteed or endorsed by the publisher.
